# A New Method to Develop the Primate Model of Knee Osteoarthritis With Focal Cartilage Defect

**DOI:** 10.3389/fbioe.2021.727643

**Published:** 2021-11-04

**Authors:** Xin Bi, Tao Li, Min Li, Shutian Xiang, Junhong Li, Bin Ling, Zhaoxiang Wu, Zhong Chen

**Affiliations:** ^1^ Department of Orthopaedic and Trauma Surgery, The Second People’s Hospital of Yunnan Province, Kunming, China; ^2^ Department of Plastic and Reconstructive Surgery, Nanfang Hospital, Southern Medical University, Guangzhou, China; ^3^ Cadre’s Physical Examination Center, The Second People’s Hospital of Yunnan Province, Kunming, China; ^4^ Department of Medical Imaging, The Second People’s Hospital of Yunnan Province, Kunming, China; ^5^ Department of Intensive Care Unit, The Second People’s Hospital of Yunnan Province, Kunming, China; ^6^ Department of Emergency Surgery, The Second People’s Hospital of Yunnan Province, Kunming, China

**Keywords:** knee, osteoarthritis, animal models, rhesus macaques, modified hulth method, cartilage injury

## Abstract

**Objective:** Osteoarthritis (OA) is a common degenerative joint disease, and animal models have proven pivotal in investigating this disease. This study aimed to develop a primate model of OA that may be more relevant to research studies on OA in humans.

**Method:** Twelve female rhesus macaques were randomly divided into three groups. Four animals were untreated (Control group); four were subjected to the modified Hulth method, involving cutting of the anterior and posterior cruciate ligaments, and transecting the meniscus (Hulth group); and four were subjected to the modified Hulth method combined with cartilage defect (MHCD group). Each primate was subjected to motor ability tests, and underwent arthroscopic, radiographic, morphological, and pathological observation of the knee joints at various times for up to 180 days.

**Results:** Motor ability on Day 180 was significantly lower in the MHCD group than in the Control (*p*＜0.01) and Hulth (*p*＜0.05) groups. Radiographic and morphological examination showed that the severity of knee joint deformity and articular cartilage injury were greater in the MHCD group than in the other groups. Pathological examination showed that cartilage thickness was significantly lower in the MHCD group than in the other groups at the same time points. The Mankin score on Day 180 was markedly higher in the MHCD group than in the Hulth (*p*＜0.05) and Control (*p*＜0.001) groups.

**Conclusion:** The MHCD model of OA closely resembles the pathophysiological processes of spontaneous knee OA in humans. The time required to develop knee OA is shorter using the MHCD model than using the Hulth method.

## Introduction

Osteoarthritis (OA) is a common degenerative joint disease that adversely affects the quality of life of millions of people around the world (Johnson and Hunter, 2014; Glyn-Jones et al., 2015). Symptoms of OA include persistent joint pain, swelling, stiffness, and limited range of motion, accompanied by progressive cartilage degradation, subchondral bone sclerosis, osteophyte formation, and synovium inflammation (Goldring and Goldring, 2010; Sokolove and Lepus, 2013). OA commonly affects the hands, feet, spine, and large weight-bearing joints, particularly the knee (Michael et al., 2010). Knee OA (KOA) is a major cause of physical disability and morbidity, imposing enormous medical and socioeconomic burdens on individuals and society (Alkan et al., 2014; Cross et al., 2014; Hawker et al., 2014). Factors associated with the development of KOA include age, strain, trauma, obesity, and genetic factors; the incidence of KOA does not differ significantly according to race or geographical location (Blagojevic et al., 2010; Guilak, 2011). To date, however, the exact causes and pathogenesis of KOA remain largely unknown, and no treatments have been found to effectively inhibit KOA progression and/or repair injured cartilage.

Animal models are an essential tool to explore the pathogenesis or KOA and to develop effective therapies (Lampropoulou-Adamidou et al., 2014). Various experimentally induced and spontaneous KOA models have been developed in several animal species, including mice, rats, rabbits, guinea pigs, dogs, sheep, goats, and horses (Kuyinu et al., 2016; Cope et al., 2019). However, the patterns of disease, particularly chronic degenerative diseases, in these animals differ markedly from those in humans (Teeple et al., 2013; Mccoy, 2015; Thysen et al., 2015). Because nonhuman primates are phylogenetically close to humans, they make powerful experimental models for studying human diseases. Monkeys, such as rhesus and cynomolgus macaques, are particularly appropriate for studying the molecular processes innate to OA development and progression, as they naturally develop OA at rates similar to those observed in humans (Bailey et al., 2014). To date, however, few KOA models have been developed in monkeys and other primates.

Surgical models of KOA developed in animals involve transaction of the anterior cruciate ligament (ACL), meniscectomy, meniscus injury, and focal cartilage defect (Gregory et al., 2012). These methods are rapid and reproducible, and are similar to the disease-initiating events and pathology of human KOA (Fang and Beier, 2014). One of the most common surgical methods used to induce OA is the Hulth method, which involves cutting the ACL and posterior cruciate ligament (PCL), and transecting the meniscus (Zhou et al., 2018). Compared with other models, KOA induced by the Hulth method is more similar to the spontaneous induction of OA observed in humans. However, the Hulth method may take a long time to produce the same degree of joint damage as a naturally degenerative model. To develop an OA model in which spontaneous development of OA is similar to that in humans, but more rapid and effective than the Hulth model, we explored a potential animal model of KOA based on rhesus macaques. This model involved using a modified Hulth method combined with focal cartilage defect (MHCD). The feasibility and validity of the MHCD method were assessed in comparison with the Hulth model.

## Materials and Methods

### Ethics Statement

All animal procedures were approved by the Institutional Animal Care and Use Committee of The Second People’s Hospital of Yunnan Province and the Kunming Institute of Zoology. CAS (SCXK(滇)K2017-0008). All animals were treated according to the guidelines of the Association for Assessment and Accreditation of Laboratory Animal Care International (AAALAC) for the ethical treatment of primates.

### Animals

Twelve female specific pathogen-free rhesus macaques (*Macaca mulatta*), aged 12.5–13.9 years and weighing 6.7–8.3 kg, were provided by the Kunming Institute of Zoology of the Chinese Academy of Sciences. The monkeys were housed individually in cages measuring 2.2 m (H) × 2.0 m (W) × 1.5 m (D) and kept in an environment with a 12 h light/dark cycle, a temperature of 22–24°C, and relative humidity of 45–55%. They were fed a standard nonhuman primate diet, and both food and water were available *ad libitum*. Additionally, the monkeys were allowed to move freely for 6–8 h each day in a spacious activity room (12.5 × 10 × 6 m) equipped with a small rockery, swings, and ball games. Occasionally, videos and music were played to relax the monkeys. The animal care staff, as well as the study staff, provided the monkeys with positive interactions.

### Grouping and Modeling

The 12 rhesus macaques were randomly divided into three groups of four. Monkeys in the Control group did not undergo surgery; monkeys in the Hulth group underwent unilateral knee surgery during which the ACL and PCL were cut and the meniscus was transected; and monkeys in the MHCD group underwent unilateral knee surgery using the MHCD method.

**FIGURE 1:**
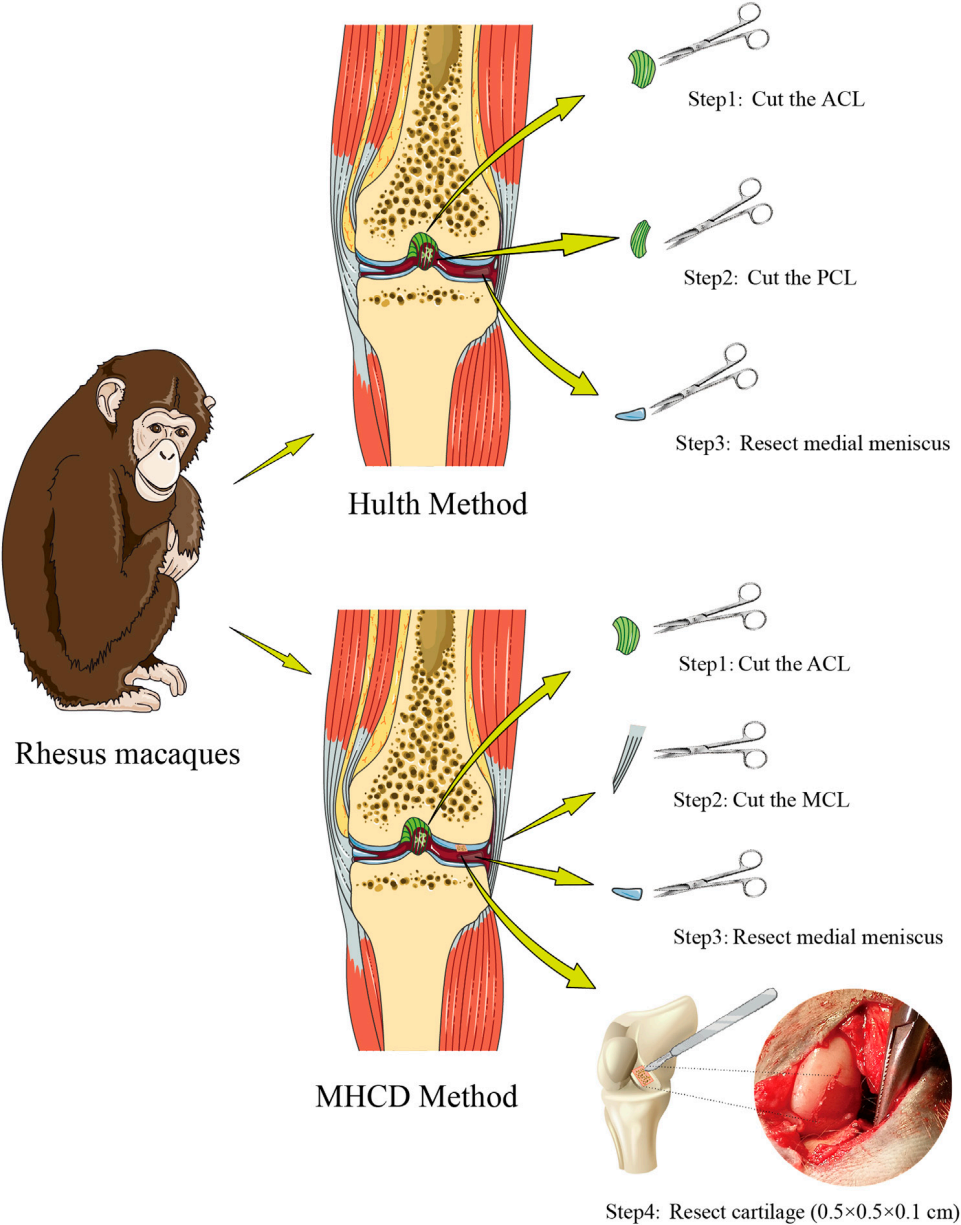
Scheme showing the processes of modeling knee osteoarthritis in rhesus macaques using the Hulth and MHCD methods. Abbreviations: MHCD, modified Hulth combined with cartilage defect; ACL, anterior cruciate ligament; PCL, posterior cruciate ligament; MCL, medial collateral ligament.

The monkeys were anesthetized by intramuscular injection of ketamine (0.2 mg/kg; Virbac, France) and fixed in a supine position on the surgical table. The site of incision on the proximal left leg was marked, and a tourniquet was applied. Anterior medial and anterior lateral approaches to the knee joint were made (incisions 4 cm in length) to enable exploration of the knee joints. Operative exploration indicated that the ACL, meniscus, articular cartilage, and other structures were all good. Subsequently, monkeys randomized to the Hulth group underwent transection of the ACL and PCL, and resection of the medial meniscus. By contrast, monkeys in the MHCD group underwent transection of the ACL, the medial collateral ligament (MCL), and the medial meniscus, along with resection of full-thickness cartilage (0.5 × 0.5 × 0.1 cm) in the weight-bearing area of the medial femoral condyle; the procedure was carried using a No. 15 blade scalpel (Jinzhong, Shanghai, China) (Figure 1). The articular cavity and incision were flushed with saline and closed with 3-0 absorbable sutures (3L Medical Products Co, Nanchang, China), followed by external fixation with a plaster cast. A soft-padded bandage was placed over the limb and maintained for 2 weeks. Starting within 3 days post-operation, cefodizime sodium (25 mg/kg; Suzhou Chung Hwa Chemical & Pharmaceutical Industrial Co, Suzhou, China) was injected intramuscularly once every 12 h to prevent infection, and parecoxib (0.8 mg/kg; Pfizer, United States) was injected intramuscularly once daily to relieve pain. Animals were monitored daily.

### Motor Ability Test

To evaluate the effect of pain caused by KOA on the motor ability of monkeys, all monkeys were monitored by video surveillance (Hikvision, China) from Day 3 to Day 180. The time for which each monkey walked and/or ran during each 24 h period was recorded.

### Radiographic Examination

On Days 60, 120, and 180, all animals were anesthetized with 10% chloral hydrate and subjected to radiographic evaluation. X-ray images of the knee joint were taken using an Axiom Multix M radiographic unit (Siemens, Germany). Magnetic resonance imaging (MRI) (Philips, the Netherlands) was also performed to assess changes in articular cartilage. X-ray images and MR images were evaluated by two radiologists and by one specialized orthopedic surgeon independently. One observer (orthopedic surgeon) evaluated all MR images twice with a 3-months interval between the two reading sessions.

### Arthroscopic Examination

Following anesthetization with 10% chloral hydrate on Day 120, the monkeys underwent arthroscopic examination (Stryker, United States) of the targeted knee joint to obtain pictures of articular surface injury.

### Morphological Examination

All animals were euthanized by administration of intensive anesthesia 180 days after the operation. The monkeys were placed in the supine position and the sites of the surgical areas were shaved. Incisions over the knee joint were made, the knee joint cavity was exposed to collect the fluid, and the articular cartilage surface was examined. Articular cartilage of the medial femoral condyle was cut from the excised knee joints and stored at −20°C before analysis.

### Histological Examination

Cartilage specimens were fixed with 4% paraformaldehyde (Solarbio Beijing, China) for 48 h at 4°C, decalcified in 20% EDTA solution (Merck, Germany), dehydrated through graded ethanol solutions, embedded in paraffin, and sliced into 5-μm sections. Sections were stained with hematoxylin and eosin (H&E; Solarbio), Safranin O (Solarbio), and toluidine blue (Solarbio), and then sealed with neutral gum. Finally, the specimens were assessed under a light microscopy (Nikon, Japan). Five slices of each articular cartilage specimen were selected, and KOA was evaluated by measuring the Mankin score(Van Der Sluijs et al., 1992).

### Enzyme-Linked Immunosorbent Assay

Synovial fluid was aspirated from the knee joints of all monkeys on Day 120. The concentrations of interleukin-1β (IL-1β), transforming growth factor-β1 (TGF-β1), and matrix metalloproteinase-13 (MMP-13) in these specimens were measured using commercially available kits (Human Quantikine ELISA kits; R&D Systems).

### Statistical Analysis

All data are presented as mean ± standard deviation (SD) and were analyzed using the SPSS 13.0 statistical package. Data distributed normally were analyzed using a *t* test, whereas non-normally distributed data were analyzed using the Mann-Whitney U test. *p*-values <0.05 were considered statistically significant.

## Results

### Baseline Characteristics and Intraoperative Condition of Rhesus Macaques

**FIGURE 2:**
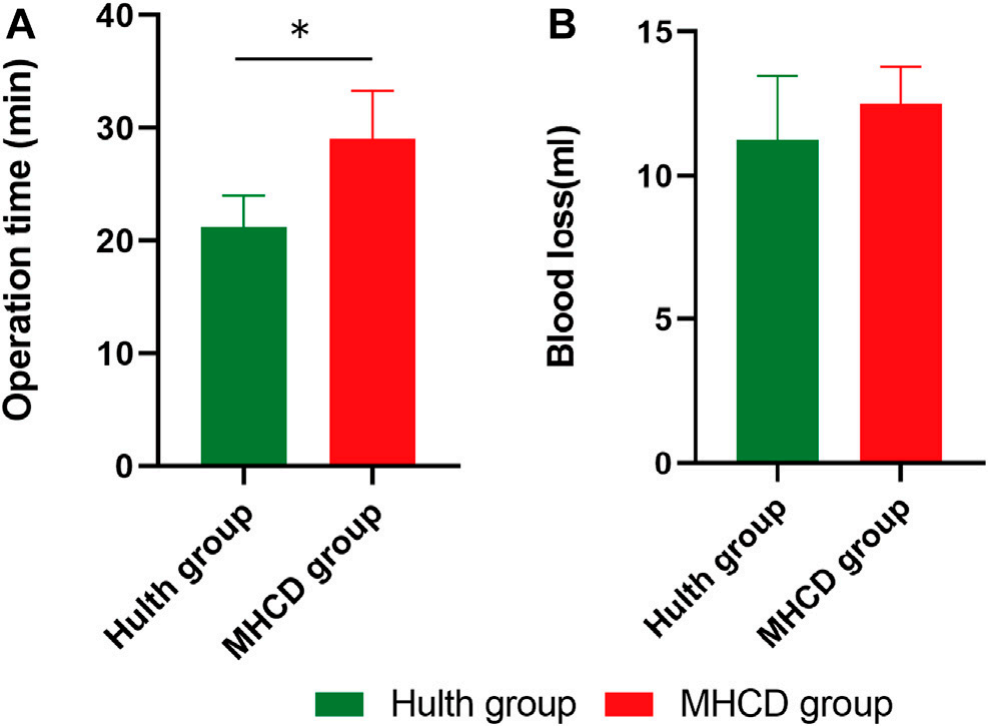
Evaluation of operation time **(A)** and blood loss **(B)** in rhesus macaques subjected to the Hulth and MHCD methods. *p < 0.05. Data are presented as the mean ± SD.

There was no significant difference between the Control, Hulth, and MHCD groups with respect to age, body weight, knee circumference, and crown sacral length (*p* > 0.05) (Table 1). Operation time was longer for the MHCD group than for the Hulth group (*p* < 0.05) (Figure 2A), but there was no significant difference in blood loss (*p* > 0.05) (Figure 2B). None of the monkeys experienced infection or died.

**TABLE 1:** Baseline characteristics of the three groups of rhesus macaques ( $\bar{x}$ ±s)

Groups	Age (year)	Weight (kg)	Knee circumference (cm)	Crown sacral length (cm)
Control group	12.93 ± 1.24	6.43 ± 0.28	15.29 ± 0.47	46.12 ± 1.36
Hulth group	13.09 ± 1.20	6.45 ± 0.30	15.20 ± 0.50	46.30 ± 1.51
MHCD group	13.16 ± 1.58	6.37 ± 0.34	15.50 ± 0.85	46.48 ± 1.71
F	1.333	2.809	2.363	0.506
P	0.311	0.113	0.150	0.619

### Analysis of Motor Ability

**FIGURE 3:**
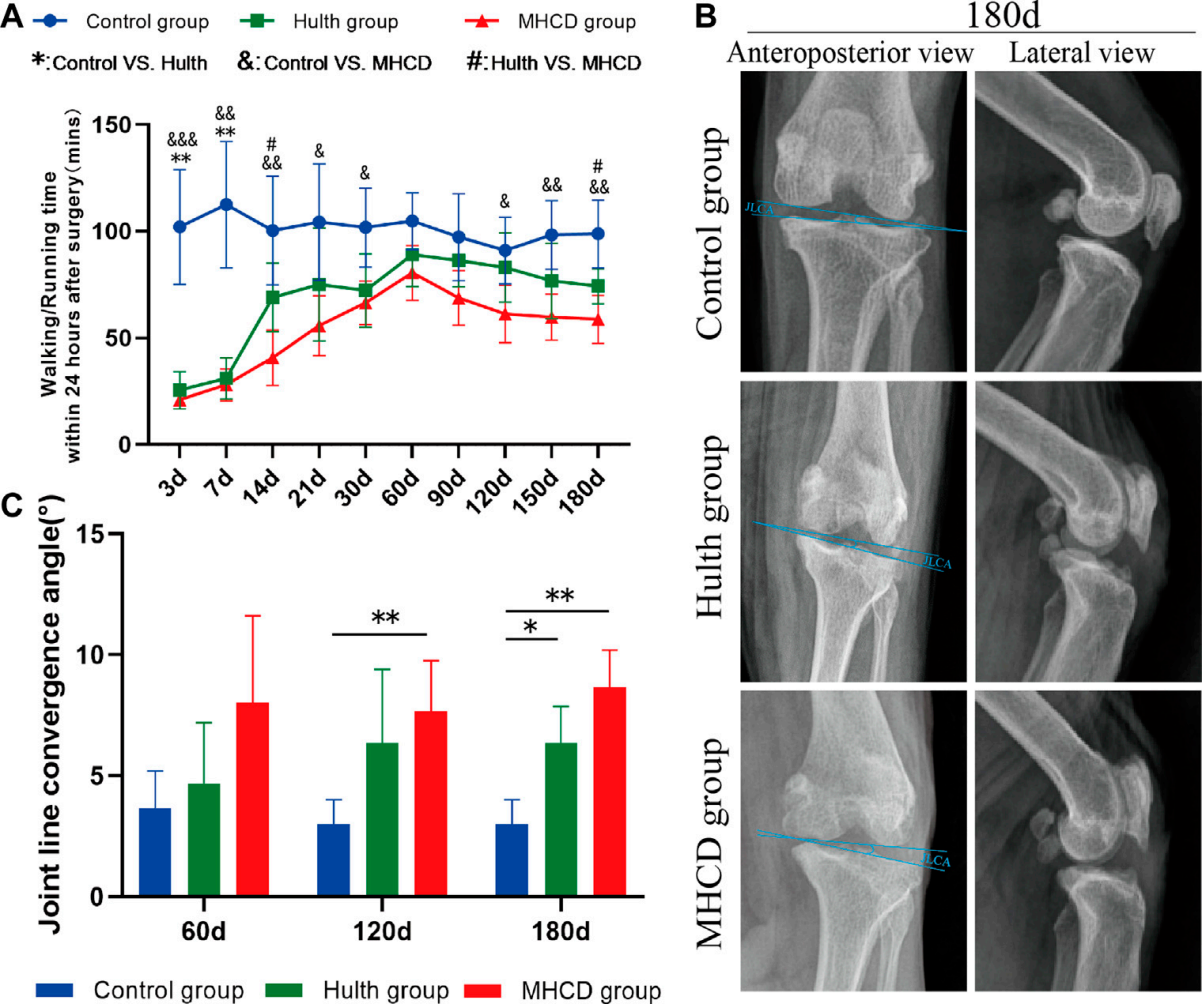
Evaluation of motor activity and postoperative X-rays in the three groups of rhesus macaques. **(A)** Motor activity within 24 h after surgery. **(B)** Representative X-rays of the knee joints on Day 180. **(C)**. Changes in joint convergence angles of the left knee. *p < 0.05, **p < 0.01, ***p < 0.001. Data are presented as the mean ± SD.

Compared with the Control group, motor ability at 3 and 7 days post-surgery was significantly lower in the Hulth (*p* < 0.01 on both days) and MHCD (*p* < 0.001 on Day 3, *p* < 0.01 on Day 7) groups due to postoperative pain. Removal of the plaster cast led to rapid relief of pain in the Hulth group, with motor ability being significantly greater than that of the MHCD group on Day 14 (*p* < 0.05). By contrast, the motor ability of the MHCD group was significantly lower than that of the Control group on Days 14 (*p* < 0.01), 21 (*p* < 0.05), and 30 (*p* < 0.05). Subsequent progression of KOA after surgery in the MHCD group resulted in gradual weakening of motor ability, which was significantly lower in the MHCD than in the Control group on Days 120 (*p* < 0.05), 150 (*p* < 0.01), and 180 (*p* < 0.01). Interestingly, motor ability on Day 180 was significantly higher in the Hulth than in the MHCD group (*p* < 0.05), but did not differ significantly between the Hulth and Control groups (Figure 3A).

### Radiographic Analysis of the Knee Joint

**FIGURE 4:**
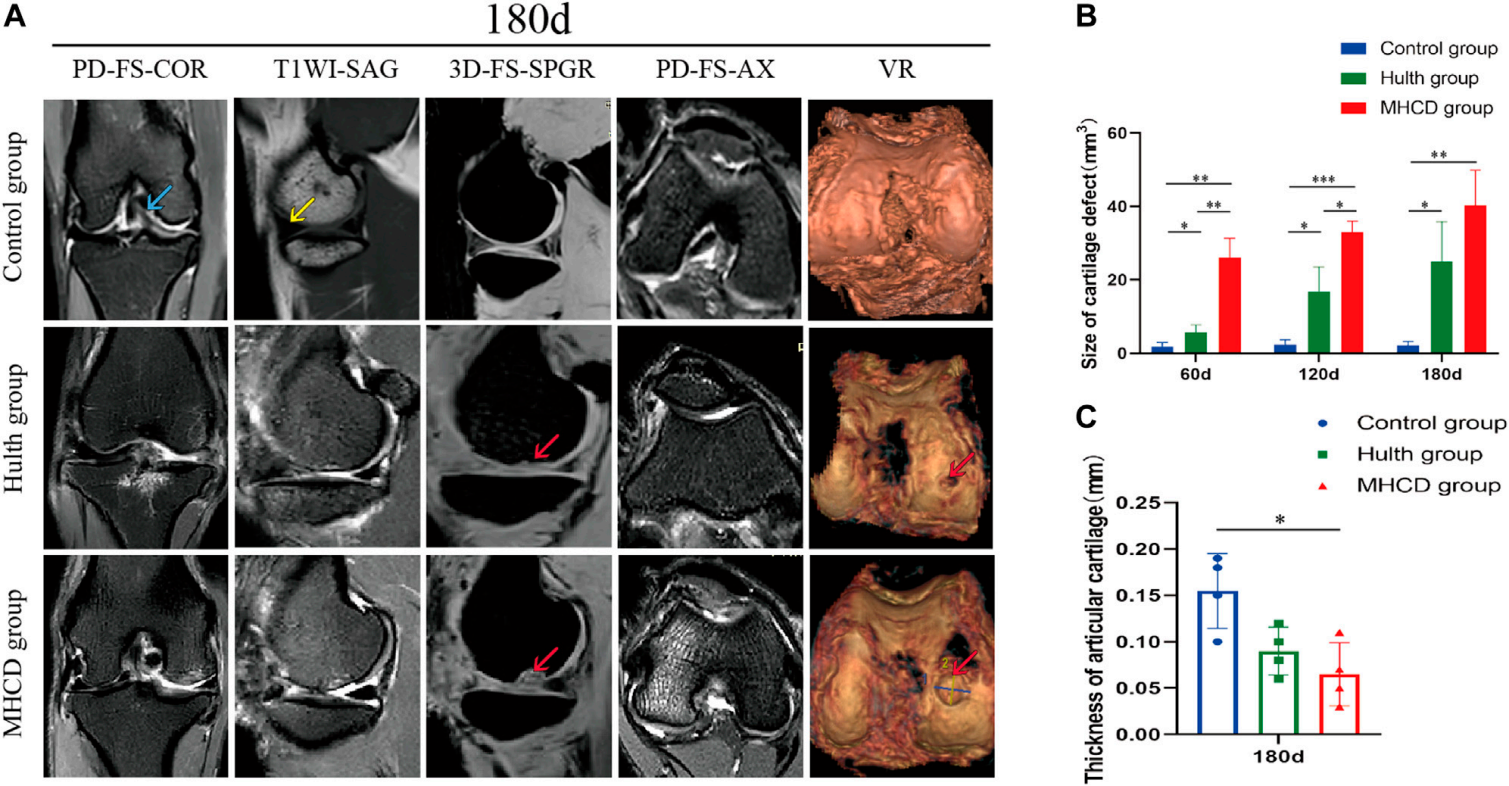
MRI findings of the knee joints of the three groups of rhesus macaques. **(A)** Representative MRI findings of the knee joints on Day 180. **(B)** Sizes of articular cartilage defects on Days 60, 120, and 180. **(C)** Thicknesses of articular cartilage in the weight-bearing part of the knee joint on Day 180. (blue arrows, ACL; yellow arrows, medial meniscus; red arrows, cartilage defects). *p < 0.05, **p < 0.01, ***p < 0.001. Data are presented as the mean ± SD.

After surgery, monkeys showed a reduced knee joint space and other gradual changes associated with KOA (Figure 3B and Figure 4A). X-rays of animals in the MHCD group showed collapse and sclerosis of the articular surface, deformity of the knee joint, and narrowing of the joint space, whereas X-rays of animals in the Hulth group showed sclerosis of the articular surface without collapse. By contrast, X-rays of the Control group showed knee joints with smooth appearance and regular surfaces (Figure 3B). On Days 120 and 180, the joint line convergence angle (JLCA) in the MHCD group was significantly greater than that in the Control group (*p* < 0.01), whereas, on Day 180, the JLCA was more extensive in the Hulth group than in the Control group (*p* < 0.05) (Figure 3C).

MRI of animals in the Hulth and MHCD groups showed slight increases in suprapatellar capsule and articular cavity effusion, medial subluxation of the patella, and injury to the articular cartilage of the medial femoral condyle (Figure 4A). Monkeys in the Hulth group showed subchondral cystic lesions in the medial femoral condyle, whereas animals in the MHCD group showed cartilage and subchondral bone damage in the medial tibial condyle, as well as joint space narrowing. The size of the cartilage defect was significantly larger in the MHCD than in the Control group on Days 60 (*p* < 0.01), 120 (*p* < 0.001), and 180 (*p* < 0.01), and was also significantly larger in the Hulth than in the Control group on Days 60, 120, and 180 (*p* < 0.05 each). In addition, the size of the cartilage defect was significantly larger in the MHCD than in the Hulth group on Days 60 (*p* < 0.01) and 120 (*p* < 0.05) (Figure 4B). On Day 180, the articular cartilage was thicker in the Control group than in the MHCD group (Figure 4C).

### Morphological Observation of the Knee Joint

**FIGURE 5:**
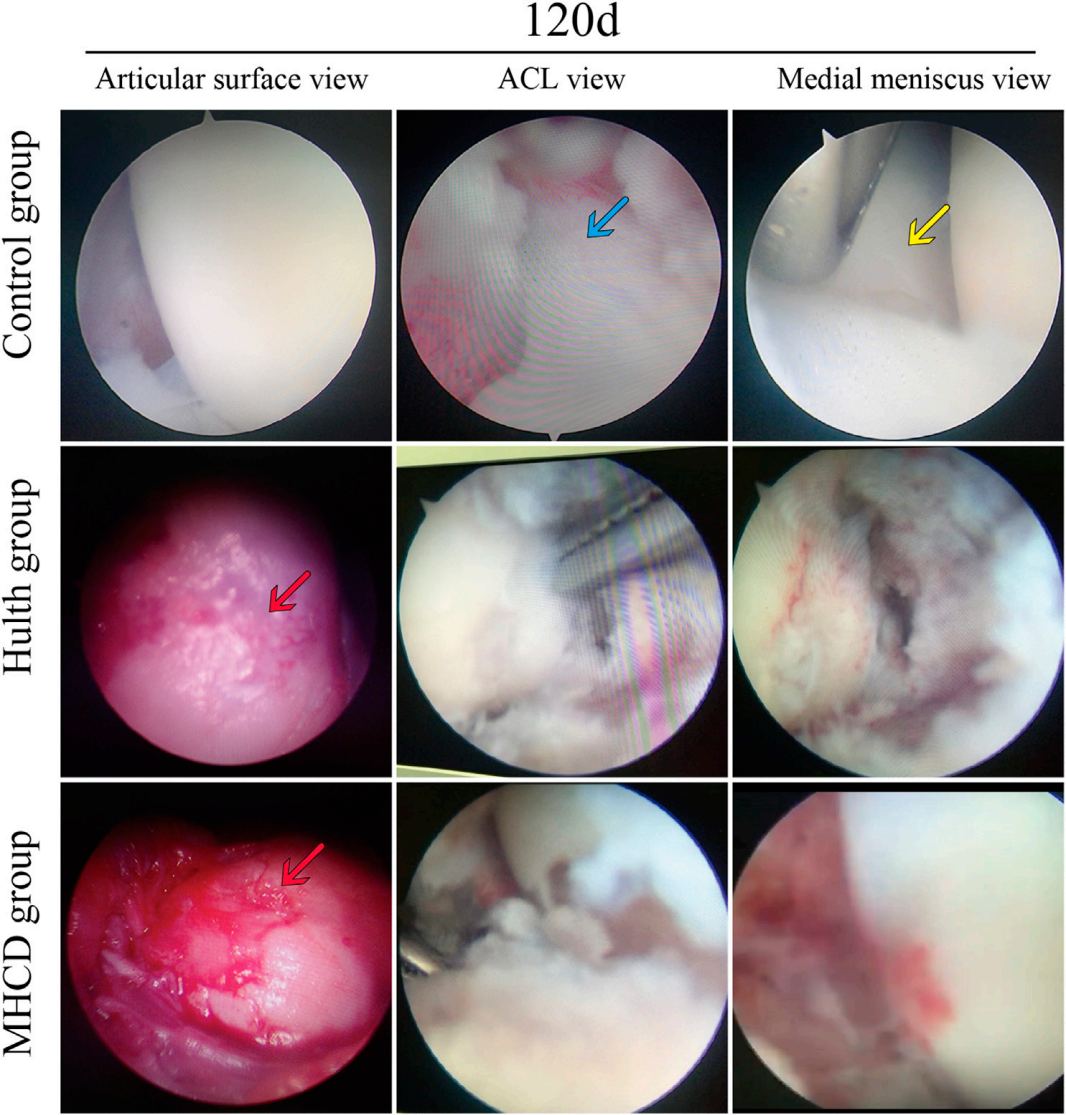
Representative arthroscopic findings of the knee joints of rhesus macaques from the Control, Hulth, and MHCD groups on Day 120 (blue arrows, ACL; yellow arrows, medial meniscus; red arrows, cartilage defects).

**FIGURE 6:**
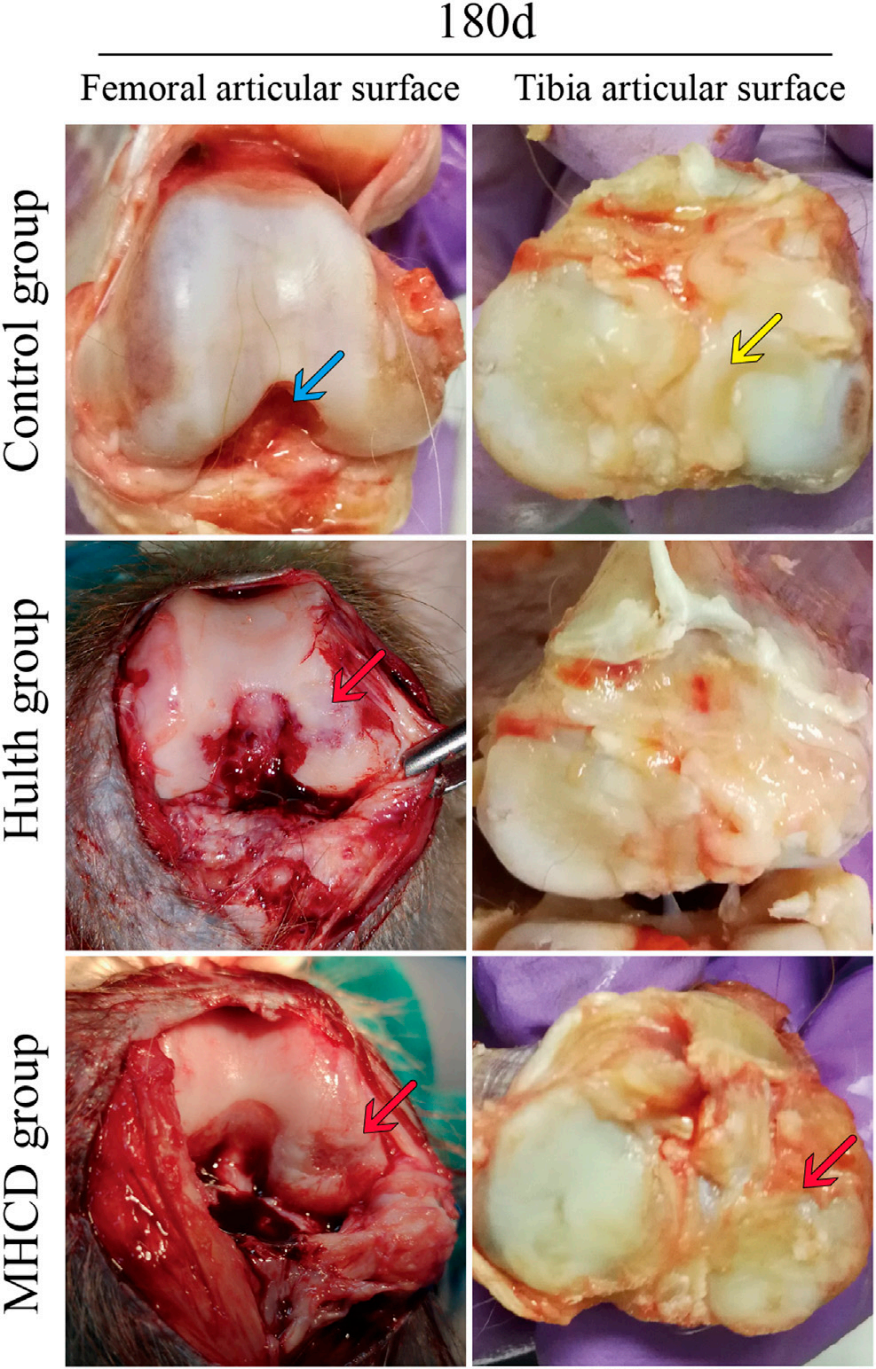
Gross observations on Day 180, showing abrasion of the weight-bearing surface of the medial femoral condyle and femoral posterior condylar cartilage in the Hulth group, but more serious abrasion in the MHCD group (blue arrows, ACL; yellow arrows, medial meniscus; red arrows, cartilage defects).

Arthroscopic views of the knee joints and the gross appearance of the femoral condyles and tibial plateaus in each group are shown in Figure 5. Arthroscopy showed that the articular cartilage in the Control group was smooth, and that the ACL and medial meniscus were present. By contrast, arthroscopy of the knees in the Hulth and MHCD groups showed absence of the ACL and medial meniscus, and wear of the medial femoral condyle cartilage. Gross examination revealed abrasion of the weight-bearing surface of the medial femoral condyle and of the femoral posterior condylar cartilage in the Hulth group, but more serious abrasion in the MHCD group. In addition, significant cartilage degeneration and cartilage thinning of the tibial plateau were evident in the MHCD group, but not in the Control and Hulth groups (Figure 6).

### Histopathologic Evaluation of Knee Joint

**FIGURE 7:**
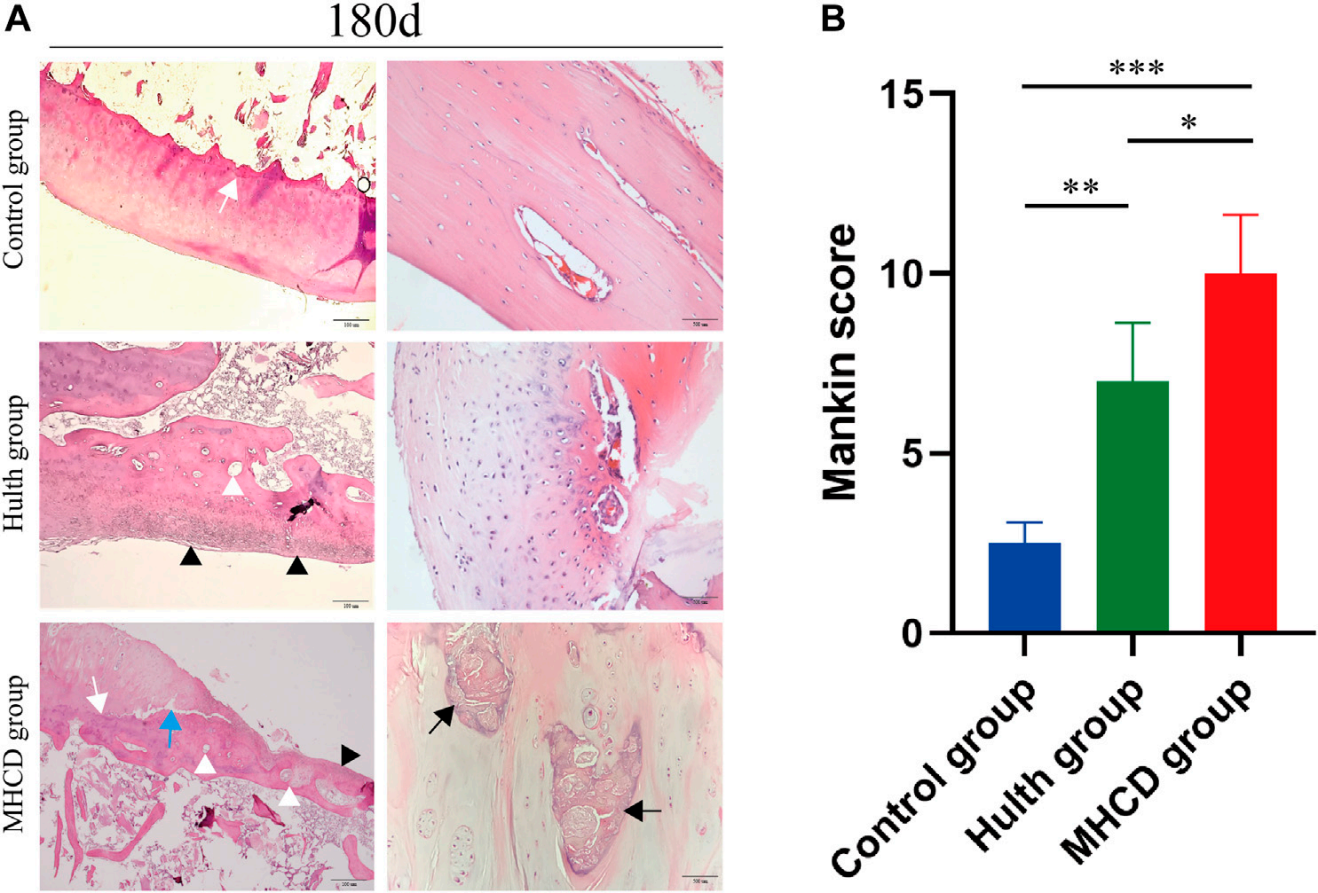
Pathological morphology of articular cartilage 180 days after surgery. **(A)** Representative images showing H&E staining of the weight-bearing surface of the medial femoral condyle and femoral posterior condylar cartilage from the Control, Hulth, and MHCD groups. (White arrow: tidemark, White arrowhead: subchondral cyst, Black arrow: sclerosis, Black arrowhead: fibrocartilage mineralization, Blue arrow: degradation of cartilage matrix) **(B)** Histologic scores of the cartilage lesions in the three groups. *p < 0.05, **p < 0.01, ***p < 0.001. Data are presented as the mean ± SD.

H&E staining of samples from the Hulth and MHCD groups on Day 180 showed damaged cartilage, disorganized chondrocyte clusters, and rough cartilage surfaces (Figure 7A). In the Hulth group, the boundary of each layer was obscure, the tidal line was continuous, and the articular hyaline cartilage was replaced gradually by fibrous tissue. In the MHCD group, however, the articular cartilage surface was damaged to different degrees, the structure was disordered, cartilage destruction involved the radiation layer, the tidal line was interrupted and blurred, and the articular cartilage was almost entirely replaced by fibrous tissue. Consistent with the radiographic and gross observations, the Mankin score showed that the severity of cartilage damage was markedly higher in the Hulth group than in the Control group (*p* < 0.01), but lower than in the MHCD group (*p* < 0.05). The Mankin score was also markedly higher in the MHCD than in the Control group (*p* < 0.001) (Figure 7B).

**FIGURE 8:**
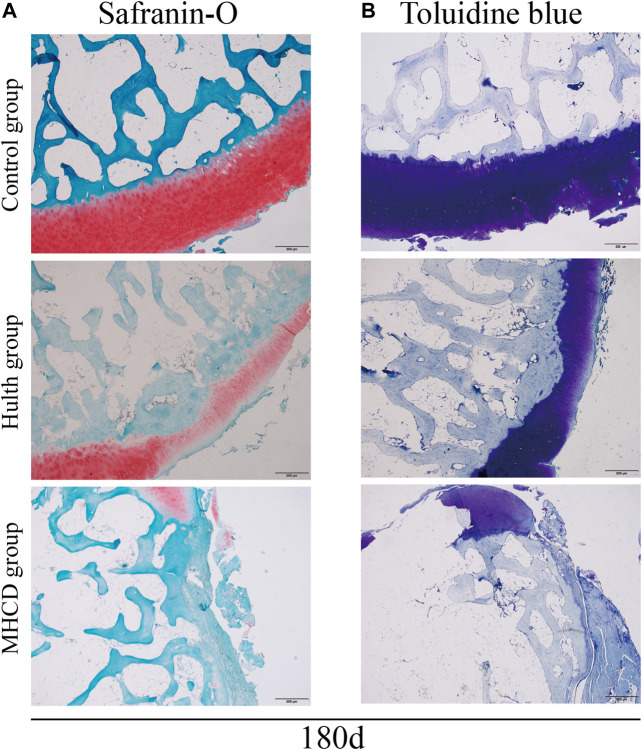
Staining of glycosaminoglycans in articular cartilage 180 days after surgery. Representative images of **(A)** Safranin O and **(B)** toluidine blue staining.

Tissue samples obtained after sacrifice on Day 180 were also stained with Safranin O, which detects proteoglycans, a major extracellular matrix component in cartilage. Safranin O staining of samples from the Control group showed a deep red color; the cartilage had a smooth surface; and the chondrocytes displayed normal morphology and were arranged in an orderly manner. By contrast, the intensity of Safranin O staining was reduced markedly in the Hulth group, as was the number of chondrocytes, which also showed disordered layers. Samples from the MHCD group showed almost complete absence of Safranin O staining, the number of chondrocytes was significantly reduced, and subchondral bone remodeling was complete (Figure 8A).

Consistent with the results of Safranin O staining, toluidine blue staining of Day 180 samples from the Control group showed a deep blue color, with chondrocytes appearing in distinct layers and arranged in an orderly manner. Samples from the Hulth group showed light blue staining of the articular cartilage, with the number of cells being significantly decreased. Samples from the MHCD group showed lower intensity of toluidine blue staining, with some of the layers showing no staining and the regions in the weight-bearing area being negative for chondrocytes (Figure 8B).

### Expression of IL-1β, TGF-β1, and MMP-13 in Synovial Fluid

**FIGURE 9:**
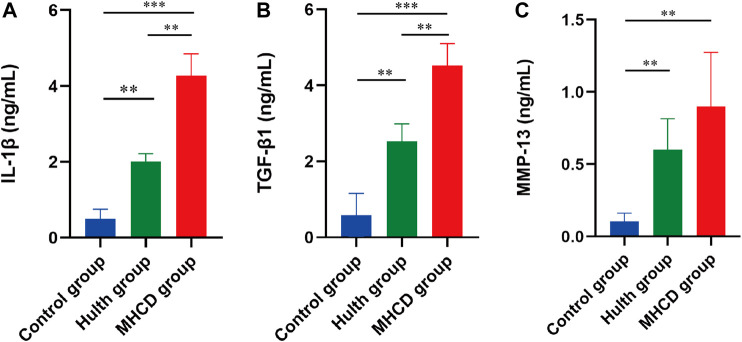
Concentrations of three representative proteins in the synovial fluid of rhesus macaques on Day 120. Concentrations of **(A)** IL-1β, **(B)** TGF-β1, and **(C)** MMP-13 were measured by ELISA. IL-1β, interleukin-1β; TGF-β1, transforming growth factor-β1; MMP-13, matrix metalloproteinase-13. *p < 0.05, **p < 0.01, ***p < 0.001. Data are presented as the mean ± SD.

Measurement of IL-1β, TGF-β1, and MMP-13 concentrations in synovial fluid on Day 120 showed that the levels of all three were significantly higher in the Hulth group than in the Control group (*p* < 0.01 each). The levels of IL-1β (*p* < 0.001), TGF-β1 (*p* < 0.001), and MMP-13 (*p* < 0.01) in the MHCD group were much higher than those in the Control group. Moreover, the levels of IL-1β (*p* < 0.01) and TGF-β1 (*p* < 0.01) were significantly higher in the MHCD group than in the Hulth group (Figures 9A–C).

## Discussion

OA is a common chronic disease characterized by articular cartilage degeneration and bone destruction, which are the main causes of joint pain and disability in patients with advanced disease (Weimer et al., 2012). Development of OA is a complex process, involving several pathological pathways and mechanisms.

Choosing an appropriate animal model of KOA is very important for assessing the mechanisms involved in the development of the disease in humans (Aigner et al., 2010). Spontaneous models are, to some extent, the closest to the natural degeneration of joints in patients with OA, but they take a long time to develop (Teeple et al., 2013). Furthermore, experimental results can be affected by many factors, thereby reducing the reliability of these models (Stone et al., 2015). Transgenic models cannot simulate the pathogenesis of human OA completely because it is driven by a combination of multiple genes and factors (Little and Hunter, 2013). Surgical methods of modeling KOA are used widely because they are rapid, simple, and highly repeatable (Gregory et al., 2012). The Hulth method induces knee joint instability and pathological changes, similar to spontaneous models of OA (Pashuck et al., 2016; Patel et al., 2016). To date, however, effective and reliable models of KOA in animals have not been quick to develop. Compared with the Hulth method, animal models of KOA induced by cartilage injury alone show significantly less damage to the biomechanical stability of the knee joint. The time needed to produce OA-related pathological changes is longer, as weight-bearing interventions are required for the injured limb (Marijnissen et al., 2002). The present study used a modified version of the Hulth method, combining it with cartilage injury to yield a KOA model that closely resembles the pathophysiological process of spontaneous KOA in humans. Furthermore, this modification led to development of KOA in a shorter time than the Hulth method.

Developing an appropriate animal model is important for exploring the pathogenesis and treatment of KOA. Various animal models have been utilized to gain insight into KOA onset and progression, and to aid development and evaluation of advanced diagnostic tools and treatments (Mccoy, 2015). For example, medial meniscectomy in male Lewis rats stimulates the pathological changes of KOA; this rat model was used to explore the effect of antiresorptive and anabolic bone therapy on post-traumatic osteoarthritis (Bagi et al., 2015). A canine spontaneous KOA model may serve as an appropriate animal model that closely mimics pathological changes in humans (Pertovaara, 2012). Moreover, this model may be used in translational pain research to test the safety and efficacy of novel analgesics. KOA was simulated in a rabbit model by transecting the PCL, providing a valuable marker of OA disease severity and progression (Gao et al., 2013). However, differences in knee morphology, biomechanics, and behavior in these animal models can make it difficult to extrapolate the findings to humans (Bailey et al., 2014). In this study, we selected rhesus macaques, a nonhuman primate, as model animals because morphologic progression of cartilage degeneration and KOA are more similar to humans than other animal models.

Although the MHCD method increased operation time compared with the Hulth method, it did not increase intraoperative blood loss. The decrease in activity during the first 30 days after surgery in both groups may be associated with postoperative pain. Interestingly, after the plaster cast was removed on Day 14, activity increased significantly in the Hulth group whereas activity in the MHCD group remained low. Damage to the knee cartilage in the MHCD group may have restricted early-stage motor activity. Although late stage (Day 180) motor activity was significantly higher in the Hulth group than in the MHCD group, activity in the Hulth and Control group was similar. The difference between the Hulth and MHCD groups may have been caused by the more significant damage to the articular cartilage in the weight-bearing area of the knee joint in the MHCD group. This result may provide indirect evidence for more rapid development of KOA after the MHCD than the Hulth method.

X-rays and MRI scans are simple and useful methods of preliminarily diagnosing KOA, with a moderate relationship reported between OA imaging features and KOA symptoms (Wu et al., 2012). Radiographic evaluation revealed pathology typical of OA in both the Hulth and MHCD groups, accompanied by severe cartilage degradation and bony changes. OA progression, however, was faster in the MHCD group than in the Hulth group. Radiological results showed that the MHCD method increased the severity of articular cartilage injury, knee joint deformity, and subchondral bone damage, suggesting that the combined method promotes pathological damage to articular cartilage and accelerates OA progression. These changes are similar to those observed for human OA and are consistent with those in previous reports of surgically-induced OA in nonhuman primates (Liu et al., 2018; Zhou et al., 2018). Furthermore, arthroscopic examination and gross morphological and cross-sectional analyses of articular surface wear suggested that pathologic degradation of the cartilage corresponded with radiographic results. Cartilage destruction in the MHCD group was more severe than in the Control and Hulth groups, as determined by the Mankin score. Joint destruction at Day 120 in the MHCD group was similar to that on Day 180 in the Hulth group, with imaging changes and morphological appearance in the MHCD group being closer to the natural degeneration observed in human KOA.

We also found that cartilage pathology was associated with increased secretion of cytokines and MMPs. Previous studies show that serum concentrations of IL-1β and TGF-β1 are higher in OA patients than in control groups, and that these concentrations are closely associated with pain (Hussein et al., 2008; Lee et al., 2011). Inflammation is involved in the pathophysiological mechanism of KOA, as evidenced by the presence of cytokines in both osteoarthritic joints and synovial fluid (Rutgers et al., 2010; Yu and Kim, 2013). These cytokines may be involved in the pathophysiology of OA by regulating expression of MMPs, which induce degradation of the extracellular matrix (Inoue et al., 2005; Haseeb and Haqqi, 2013; Lim et al., 2014). Cytokines and MMPs are involved in the development of cartilage damage (Bondeson et al., 2006; Klatt et al., 2006; Pujol et al., 2008). The present study found that the levels of IL-1β, TGF-β1, and MMP13 in synovial fluid were significantly higher in the MHCD than in the Hulth and Control groups, and were significantly higher in the Hulth than in the Control group. On Day 120, expression of cytokines and MMP-13 in synovial fluid of rhesus macaques with MHCD-induced KOA was similar to that in patients with spontaneous KOA. The inflammatory and biomechanical changes in synovial fluid were less serious animals subjected to the Hulth method.

This study had several limitations. First, due to the lack of suitable experimental animals and funds, groups of animals with spontaneous KOA and those subjected to sham operations were not included. Second, the study included a small number of animals, suggesting caution in interpreting the results. Additional studies are required to compare MHCD-induced and spontaneous models of KOA, as well as KOA in primates of different genders and ages.

In conclusion, the present study describes the development of a primate model of KOA using a modified Hulth method combined with focal cartilage defects. This model is similar to spontaneous models with respect to osteoarthritic and histopathologic grading, as well as changes in expression of inflammatory cytokines and MMPs. Furthermore, compared with the Hulth method, the MHCD method resulted in more rapid development of KOA, suggesting that the MHCD method may be a suitable model for studying the pathogenesis and treatment of OA.

## Data Availability

The original contributions presented in the study are included in the article/supplementary material, further inquiries can be directed to the corresponding authors.
